# Supplementary material for: Transcriptome Analysis Reveals Vimentin-Induced Disruption of Cell–Cell Associations Augments Breast Cancer Cell Migration
Source: Cells. 2022 Dec 13;11(24):4035. doi: 10.3390/cells11244035 (PMC9776984; doi:10.3390/cells11244035)
Supplement: Supplementary file 1 [file cells-11-04035-s001.zip › DEGs and enrichment analysis.zip!/DEGs and enrichment analysis/WT_VIMvsVector KEGG/UP/WT_VIMvsVector_UP.pathway_map.zip!/WT_VIMvsVector_UP.pathway_map/src/hsa05163.html]

hsa05163


- hsa:51764

- Up regulated genes

ENSG00000127588(3.22298284123795)

- hsa:107

- Up regulated genes

ENSG00000164742(1.87205666251591)

- hsa:6387

- Up regulated genes

ENSG00000107562(4.08892060137154)

- hsa:1388

- Up regulated genes

novel.600(2.78845209317737)

- hsa:1870

- Up regulated genes

novel.507(3.55620908288126)
- hsa:1869

- Up regulated genes

ENSG00000101412(1.977544660594)

- hsa:107

- Up regulated genes

ENSG00000164742(1.87205666251591)

- hsa:51764

- Up regulated genes

ENSG00000127588(3.22298284123795)

- hsa:51764

- Up regulated genes

ENSG00000127588(3.22298284123795)

- hsa:107

- Up regulated genes

ENSG00000164742(1.87205666251591)

- hsa:51764

- Up regulated genes

ENSG00000127588(3.22298284123795)

Close
